# Adaptive L_1/2_ Shooting Regularization Method for Survival Analysis Using Gene Expression Data

**DOI:** 10.1155/2013/475702

**Published:** 2013-12-15

**Authors:** Xiao-Ying Liu, Yong Liang, Zong-Ben Xu, Hai Zhang, Kwong-Sak Leung

**Affiliations:** ^1^Faculty of Information Technology & State Key Laboratory of Quality Research in Chinese Medicines, Macau University of Science and Technology, Macau 999078, China; ^2^Faculty of Science, Xi'an Jiaotong University, Xi'an 710000, China; ^3^Department of Computer Science and Technology, The Chinese University of Hong Kong, Hong Kong 999077, China

## Abstract

A new adaptive L_1/2_ shooting regularization method for variable selection based on the Cox's proportional hazards mode being proposed. This adaptive L_1/2_ shooting algorithm can be easily obtained by the optimization of a reweighed iterative series of L_1_ penalties and a shooting strategy of L_1/2_ penalty. Simulation results based on high dimensional artificial data show that the adaptive L_1/2_ shooting regularization method can be more accurate for variable selection than Lasso and adaptive Lasso methods. The results from real gene expression dataset (DLBCL) also indicate that the L_1/2_ regularization method performs competitively.

## 1. Introduction

In the study of the dependence of survival time *T* on covariances *X*, the Cox's proportional hazards model [1, 2] is the most widely used model in survival analysis. Suppose the dataset has a sample size of *n* to study survival time *T* on covariate *X*, we use the data form of (*t*_1_, *δ*_1_, *X*_1_),…, (*t*_*n*_, *δ*_*n*_, *X*_*n*_) to represent the individual's sample, where *δ* is the censoring indicator, the *t*_*i*_ denotes the survival time if *δ*_*i*_ = 1 or otherwise censoring time.

By the Cox's proportional hazards model, the hazard function can be defined as

(1) $$\begin{array}{c} h\left(t\;|\;\beta \right)=h_{0}\left(t\right)\exp\left(\beta^{T}X\right), \end{array}$$

where baseline hazard function *h*_0_(*t*) is unspecified or unknown and *β* = (*β*_1_, *β*_2_,…, *β*_*p*_) is the regression coefficient vector of *p* variables.

The Cox's partial log-likelihood is expressed as

(2) $$\begin{array}{c} l\left(\beta \right)=\sum_{i=1}^{n}\delta_{i}\left\{x_{i}^{T}\beta -\log\left(\sum_{j\in R_{i}}\exp\left(x_{j}^{T}\beta \right)\right)\right\}, \end{array}$$

where *R*_*i*_ = {*j* ∈ 1,…, *n*,  *t* > *t*_*i*_} denotes ordered risk set at time *t*_*i*_; *t*_*i*_ represents failure time.

In practice, not all the *n* covariates may contribute to the prediction of survival outcomes: some components of *β* may be zero in the true model. To select important variables under the proportional hazards model (2), Tibshirani [3], Fan and Li [4], and Zhang and Lu [5] proposed to minimize the penalized log partial likelihood function as

(3) $$\begin{array}{c} -\frac{1}{n}l\left(\beta \right)+\lambda \sum_{j=1}^{p}P\left(\beta_{j}\right). \end{array}$$

The standard regularization algorithm cannot directly be applied for nonlinear Cox model to obtain parameter estimates. Therefore, Tibshirani [3] and Zhang and Lu [5] proposed iterative procedure to transform the Cox's partial log-likelihood function (2) to linear regression problem through an iteratively Newton-Raphon update. Here we follow the approach of Zhang and Lu [5]: define the gradient vector ∇*l*(*β*) = −∂*l*(*β*)/∂*β* and the Hessian matrix ∇^2^ *l*(*β*) = −∂*l* ^2^(*β*)/∂*β*∂*β* ^*T*^, then apply the Choleski decomposition to obtain *X* ^*T*^ = {∇^2^ *l*(*β*)}^1/2^, and generate the pseudoresponse vector *Y* = (*X* ^*T*^)^−1^{∇^2^ *l*(*β*)*β* − ∇*l*(*β*)}. Then Zhang and Lu [5] suggested an optimization problem with the penalty function:

(4) $$\begin{array}{c} \hat{\beta }=\text{arg min}\left\{{\left(Y-X\beta \right)}^{T}\left(Y-X\beta \right)+\lambda \sum_{j=1}^{p}P\left(\beta_{j}\right)\right\}.\; \end{array}$$

The Lasso penalty is *P*(*β*_*j*_) = |*β*_*j*_|, which shrinks small coefficients to zero and hence results in a sparse representation of the solution. However, estimation of large *β*'s may suffer from substantial bias in *λ* if chosen too big and may not be sufficiently spare if *λ* is selected too small. Hence, Fan and Li [4] proposed the smoothly clipped absolute deviation (SCAD) penalty, which avoids excessive penalties on large coefficients and enjoys the oracle properties. The adaptive penalty is *P*(*β*_*j*_) = |*β*_*j*_|/|*β*_*j*_′|, where the weights 1/|*β*_*j*_′| are chosen adaptively by data. The values chosen for 1/|*β*_*j*_′| are crucial for guaranteeing the optimality of the solution.

The above-mentioned series of Lasso methods were based on the L_1_ penalty. Xu et al. [6, 7] and Liang et al. [8] have proposed L_1/2_ regularization method which has the L_1/2_ penalty *P*(*β*_*j*_) = |*β*_*j*_|^1/2^. The theoretical analyses and experiments show that the L_1/2_ regularization is more effective than Lasso both in theory and practice. In this paper, we investigate the adaptive L_1/2_ shooting regularization to solve the Cox model.

The rest of the paper is organized as follows.  Section 2 describes an adaptive L_1/2_ shooting regularization algorithm to obtain estimates from the Cox model.  Section 3 evaluates our method by simulation studies and application to real gene expression dataset (DLBCL). Finally we give a brief discussion.

## 2. Adaptive L_1/2_ Shooting Regularization Method for the Cox Model

The log partial likelihood function of the Cox model with the L_1/2_ penalty is

(5) $$\begin{array}{c} \beta_{1/2}=\text{arg min}\left\{\frac{1}{n}\sum_{i=1}^{n}{\left(Y_{i}-X_{i}^{T}\beta \right)}^{2}+\lambda \sum_{i=1}^{p}{\left|\beta_{i}\right|}^{1/2}\right\}, \end{array}$$

where *λ* is the tuning parameter.

In this section, we proposed the adaptive L_1/2_ shooting algorithm to optimize the Cox model in an approximate linear form. The following is the complete algorithm procedure.

**Step 1**

Initial coefficients value *β* ^0^ = (*β*_1_^0^, *β*_2_^0^,…, *β*_*p*_^0^) = (1,1,…, 1) and *t* = 0.

**Step 2**

Compute ∇*l*, ∇^2^ *l*, *X*, *Y*, and $\omega_{j}=1/\sqrt{\beta_{j}^{t}}$ based on *β*_*j*_^*t*^ (1 ≤ *j* ≤ *p*), define RSS = (*Y* − *Xβ*)^*T*^(*Y* − *Xβ*), *S*_*j*_ = ∂RSS/∂*β*_*j*_^*t*^ (1 ≤ *j* ≤ *p*), and write *β* ^*t*^ as (*β*_*j*_^*t*^, (*β*_−*j*_^*t*^)^*T*^)^*T*^, where *β*_−*j*_^*t*^ is the (*p* − 1)-dimensional vector consisting of all *β* ^*t*^'s other than *β*_*j*_^*t*^, let *S*_0_ = *S*_*j*_(0, *β*_−*j*_^*t*^) for each *j* = 1,…, *p*.

**Step 3**

Solve *β* ^*t*+1^ = arg min{(*Y* − *Xβ*)^*T*^(*Y* − *Xβ*) + *λ*∑_*j*=1_^*p*^|*β*_*j*_|^1/2^} (1 ≤ *j* ≤ *p*), using the L_1/2_ shooting regularization approach:

(6) βjt∗={λ·ωj−2S04xjTxj,if  S0>12λ·ωj,−λ·ωj−2S04xjTxj,if  S0<12λ·ωj,0,if  |S0|≤12λ·ωj.

**Step 4**

Solve $\beta^{t+1}=\text{arg min}\{{\left(Y-X\beta \right)}^{T}(Y-X\beta )+\lambda \sum_{j=1}^{p}\left|\beta_{j}\right|/\sqrt{\beta_{j}^{t}}\}$ (1 ≤ *j* ≤ *p*), using the modified reweighed iterative approach of the L_1_ shooting approach.

*Step  4.1.* Start with *β* ^*t*,*m*^ = (*β*_1_^*t*,*m*^, *β*_2_^*t*,*m*^,…, *β*_*p*_^*t*,*m*^) = *β* ^*t*^, set inner iteration count *m* = 0.

*Step  4.2.* At each iterative step *m*, for each *j* = 1,…, *p*, update:

(7) βjt,m+1={λ·ωj−S02xjTxj,if  S0>λ·ωj,−λ·ωj−S02xjTxj,if  S0<λ·ωj,0,if  |S0|≤λ·ωj,

where *x*_*j*_ is the *j*th column of *X*. A new estimator *β*_*j*_^*t*,*m*^ is formed after updating all *β*_*j*_'s and let *m* = *m* + 1.

*Step  4.3.* Update *ω*_*j*_ and *S*_0_ and repeat Step 4.2 until *β* ^*t*,*m*^ converge.

**Step 5**

Let *t* = *t* + 1 and update *β*_*j*_^*t*+1^ = min⁡(*β*_*j*_^*t*,*m*^, *β*_*j*_^*t*∗^) and *j* = 1,…, *p* and repeat Steps 2, 3, and 4 until *β* ^*t*+1^ does not change.

In Steps 2 and 4.3, we modify shooting algorithm with weight $1/\sqrt{\left|\beta_{j}^{t}\right|}$ based on last estimate *β* ^*t*^ at each iteratively step. It is possible that some *β* ^*t*^ become zero during the iterative procedure. So to guarantee the feasibly, we replace $1/\sqrt{\left|\beta_{j}^{t}\right|}$ with $1/\sqrt{\left|\beta_{j}^{t}+\varepsilon \right|}$ when implementing, where *ε* is any fixed positive real number. Steps 3 and 4 implement the shooting strategy of L_1/2_ penalty and the reweighed iterative strategy of L_1_ penalties, respectively.  Step 5 selects the minimum of *β* ^*t*^, which is obtained by Steps 3 and 4, to improve the converge speed of the algorithm.

This algorithm gives exact zeros for some coefficients and it converges quickly based on our empirical experience. Similarly to Theorem 3 in Fu [9], we can show that the adaptive L_1/2_ shooting regularization algorithm is guaranteed to converge to the global minimum of the log partial likelihood function of the Cox model (5).

## 3. Numerical Studies

### 3.1. Simulation Study for the High Dimensional Artificial Dataset

In this section, we compare the performance of the Lasso, the adaptive Lasso, and the adaptive L_1/2_ shooting regularization method, under Cox's proportional hazards model. The cross-validated partial likelihood (CVPL) method is used to estimate the tuning parameter *λ* in these three algorithms. In our simulation studies, we use the Gempertz model suggested by Qian et al. [10] to generate the Cox model datasets in the setting:

(8) β=(−0.7,−0.5,−0.3,−0.1,0,0,0,0,0,0,0.4,0,0,0.7︷14,0,…,0︷986︸1000).

We considered the cases with 25% and 40% of censoring and used four samples, *n* = 200, 250, 300, and 350. The simulation results obtained by the three methods reported in Table 1. Since this simulation dataset has 6 relevant features (6 nonzero coefficients) in the 1000 ones, the idealized average numbers of variables selected (the Var column) and correct zeros (the Corr column) by each method are 6 and 994, respectively. From the Var and Corr columns of Table 1, the results obtained by the L_1/2_ regularization method are obviously better than those of other methods for different sample sizes and censoring settings. For example, when *n* = 200 and the censoring is 25%, the average numbers (Var) from the Lasso, the adaptive Lasso, and the L_1/2_ regularization methods are 81.29, 41.06, and 17.79 (best). The correct zeros' numbers (Corr) of the three methods are 917.29, 962.47, and 984.28 (best), respectively. The results obtained by the L_1/2_ method are obviously close to the idealized values in the Var and Corr columns. Moreover, in the IBS (the integrated Brier score) column, the IBS's value of the Lasso, the adaptive Lasso, and the L_1/2_ shooting regularization method are 0.1502, 0.1474, and 0.1440. This means that the L_1/2_ shooting regularization method performs slight better than the other two methods for the prediction accuracy. Similar results are observed for the 40% censoring case.

As shown in the Incorr columns of Table 1, the idealized average number is 0 if the method can correctly identify all relevant variables at each run, whereas its maximal value is 6 if the method incorrectly identifies all the nonzero coefficients to zero in all runs. When the sample size is relative small (*n* = 200 and censoring rate = 25%), the average number of the incorrect zeros from the Lasso is 0.26, from the adaptive Lasso is 0.35 and from the L_1/2_ regularization shooting method is 0.42. The adaptive L_1/2_ shooting regularization method performs worse than the other two methods. When *n* increases to 350, all the three algorithms never evaluated the nonzero coefficients to zero. This means that the adaptive L_1/2_ shooting regularization method shrinks the small effect covariates to zero more easily than the Lasso and the adaptive Lasso when the sample size is relative small. Similar results are observed for the 40% censoring case.

### 3.2. Experiments on the Real Gene Expression (DLBCL) Dataset

To further demonstrate the utility of the L_1/2_ regularization shooting procedure in relating microarray gene expression data to censored survival phenotypes, we re-analyzed a published dataset of DLBCL by Rosenwald et al. [11]. This dataset contains a total of 240 patients with DLBCL, including 138 patient deaths during the followups with a median death time of 2.8 years. Rosenwald et al. [11] divided the 240 patients into a training set of 160 patients and a test set of 80 patients and built a multivariate Cox model. The variables in the Cox model included the average gene expression levels of smaller sets of genes in four different gene expression signatures together with the gene expression level of BMP6. It should be noted that in order to select the gene expression signatures, they performed a hierarchical clustering analysis for genes across all the samples (including both training and test samples). In order to compare our results with those in Rosenwald et al. [11], we used the same setting of training and test datasets in our analysis.

We applied the adaptive L_1/2_ shooting regularization method to first build a predictive model using the training data of 160 patients and all the 7399 genes as features (predictors).  Table 2 shows the GeneBank ID and a brief description of top ten genes selected by our proposed L_1/2_ regularization method. It is interesting to note that eight of these genes belong to the gene expression signature groups defined in Rosenwald et al. [11]. These three signature groups include Germinal-center B-cell signature, MHC, and lymph-node signature. On the other hand, two genes selected by the L_1/2_ method are not in the proliferation signature group defined by Rosenwald et al. [11].

Based on the estimated model with these genes, we estimated the risk scores using the method proposed by Gui and Li [12]. To further examine whether clinically relevant groups can be identified by the model, we used zero as a cutoff point of the risk scores and divided the test patients into two groups based on whether they have positive or negative risk scores (*f*(*x*) = *β* ^*T*^ *x*).

**Figure 1:**
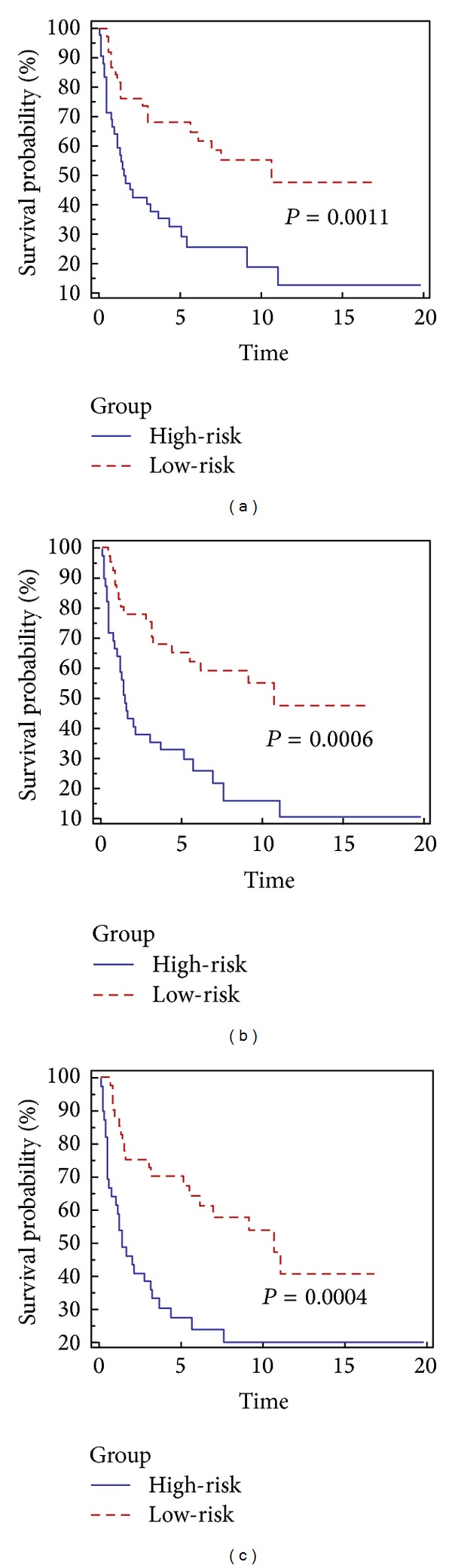
The Kaplan-Meier curves for the high- and low-risk groups defined by the estimated scores for the 80 patients in the test dataset. The scores are estimated based on the models estimated by the Lasso method (plot (a)), the adaptive Lasso method (plot (b)), and the L_1/2_ regularization shooting method (plot (c)). The maximal follow-up time is 20 years.

As a comparison, the Lasso, the adaptive Lasso, and the L_1/2_ regularization methods are validated on the test dataset of 80 patients defined in Rosenwald et al. [11], and their corresponding Kaplan-Meier curves are shown in Figure 1. In Figure 1, the horizontal coordinate is the predictive survival time (years) and the vertical coordinate is the predictive survival probabilities. The *P* value (lower the better to indicate statistical significance) of the Lasso for the test dataset is 0.0011, which is significantly larger than 0.0006 and 0.0004 of the adaptive Lasso and the L_1/2_ regularization methods. This means that lasso method performs the worst for the survival prediction compared with other two methods.

On the other hand, in order to assess how well the model predicts the outcome, we also use the idea of the integrated Brier score (IBS) for the test dataset including censored observations as our criteria. In Table 3, the IBS's value of the Lasso, the adaptive Lasso, and the adaptive L_1/2_ shooting regularization method are 0.2306, 0.2026, and 0.2017. We can see that the adaptive Lasso and the adaptive L_1/2_ shooting regularization methods perform slight better than Lasso for the prediction accuracy.

## 4. Discussion and Conclusion

In this paper, we have presented the novel adaptive L_1/2_ shooting regularization method, which is used for variable selection in the Cox's proportional hazards model. Its performance is validated by both simulation and real case studies. In the experiments, we use the high-dimensional and low-sample size dataset, with applications to microarray gene expression data (DLBCL). Results indicate that our proposed adaptive L_1/2_ shooting regularization algorithm is very competitive in analyzing high dimensional survival data in terms of sparsity of the final prediction model and predictability. The proposed L_1/2_ regularization procedure is very promising and useful in building a parsimonious predictive model used for classifying future patients into clinically relevant high-risk and low-risk groups based on the gene expression profile and survival times of previous patients. The procedure can also be applied to select important genes which are related to patient's survival outcome.

## Figures and Tables

**Table 1:** The simulation results based on the high dimensional simulated dataset by the three methods over 100 replications. The columns include the average number of the selected variable (Var), the average number of the correct zeros (Corr), the average number of the incorrect zeros (Incorr), and the integrated Brier score (IBS). (Lasso: the Lasso method, A-L: the adaptive Lasso method, and L_1/2_: the adaptive L_1/2_ shooting regularization method).

*n*	25% censoring					40% censoring			
	Method	Var	Corr (994)	Incorr (0)	IBS	Var	Corr (994)	Incorr (0)	IBS
200	Lasso	81.29	917.29	0.26	0.1502	96.38	906.83	0.31	0.1516
	A-L	41.06	962.47	0.35	0.1474	59.05	948.89	0.43	0.1503
	L_1/2_	17.79	984.28	0.42	0.1440	20.42	974.15	0.53	0.1498

250	Lasso	98.46	903.07	0.11	0.1462	148.87	883.85	0.15	0.1493
	A-L	64.10	949.46	0.17	0.1446	74.42	933.74	0.26	0.1478
	L_1/2_	27.38	972.95	0.25	0.1421	31.91	968.03	0.34	0.1458

300	Lasso	167.82	883.18	0.01	0.1448	177.50	869.83	0.03	0.1479
	A-L	72.95	932.49	0.02	0.1436	80.97	927.42	0.06	0.1459
	L_1/2_	33.45	967.12	0.03	0.1418	38.64	958.38	0.06	0.1427

350	Lasso	196.24	847.84	0.00	0.1441	204.22	834.53	0.00	0.1463
	A-L	82.80	928.07	0.00	0.1428	89.18	921.54	0.00	0.1441
	L_1/2_	37.58	959.78	0.00	0.1405	40.15	948.63	0.00	0.1412

**Table 2:** GeneBank ID and descriptions of the top 10 genes selected by the adaptive L_1/2_ shooting regularization method based on the 160 patients in the training dataset. Indicated are the gene expression signature groups that these genes belong to; Germ: Germinal-center B-cell signature, MHC: MHC class II signature, and Lymph: lymph-node signature. Genes NM_005191 and X82240 do not belong to these signature groups.

GeneBank ID	Signature	Description
NM_005191		Homosapiens CD80 molecule (CD80), mRNA
AA714513	MHC	major histocompatibility complex, class II, DR beta 5
AA598653	Lymph	osteoblast specific factor 2 (fasciclin I-like)
AA767112	MHC	major histocompatibility complex, class II, DP beta 1
LC_24433	Lymph	
AA840067	Germ	TCL1A T-cell leukemia/lymphoma 1A
X82240		Homosapiens mRNA for T-cell leukemia
AA700997	Germ	cell associated 1
AA505045	Germ	Homosapiens, clone MGC:3963 IMAGE:3621362, mRNA, complete CDs
AA805575	Germ	Thyroxine-binding globulin precursor

**Table 3:** The integrated Brier score (IBS) obtained by the Lasso, the adaptive Lasso and the adaptive L_1/2_ shooting regularization method for DLBCL dataset. (Lasso: the Lasso method; A-L: the adaptive Lasso method; L_1/2_: the adaptive L_1/2_ shooting regularization method).

	Lasso	A-L	L_1/2_
IBS	0.2306	0.2026	0.2017
